# Analysis of acute lymphoblastic leukemia drug sensitivity by changes in impedance via stromal cell adherence

**DOI:** 10.1371/journal.pone.0258140

**Published:** 2021-09-30

**Authors:** Annie Luong, Fabio Cerignoli, Yama Abassi, Nora Heisterkamp, Hisham Abdel-Azim

**Affiliations:** 1 Division of Hematology, Oncology and Bone Marrow Transplantation, Children’s Hospital Los Angeles, Los Angeles, CA, United States of America; 2 Agilent Technologies, Inc., Santa Clara, CA, United States of America; 3 Department of Systems Biology, Beckman Research Institute City of Hope, Monrovia, CA, United States of America; 4 Keck School of Medicine, University of Southern California, Los Angeles, CA, United States of America; The University of Adelaide, AUSTRALIA

## Abstract

The bone marrow is a frequent location of primary relapse after conventional cytotoxic drug treatment of human B-cell precursor acute lymphoblastic leukemia (BCP-ALL). Because stromal cells have a major role in promoting chemotherapy resistance, they should be included to more realistically model *in vitro* drug treatment. Here we validated a novel application of the xCELLigence system as a continuous co-culture to assess long-term effects of drug treatment on BCP-ALL cells. We found that bone marrow OP9 stromal cells adhere to the electrodes but are progressively displaced by dividing patient-derived BCP-ALL cells, resulting in reduction of impedance over time. Death of BCP-ALL cells due to drug treatment results in re-adherence of the stromal cells to the electrodes, increasing impedance. Importantly, vincristine inhibited proliferation of sensitive BCP-ALL cells in a dose-dependent manner, correlating with increased impedance. This system was able to discriminate sensitivity of two relapsed Philadelphia chromosome (Ph) positive ALLs to four different targeted kinase inhibitors. Moreover, differences in sensitivity of two *CRLF2*-drivenBCP-ALL cell lines to ruxolitinib were also seen. These results show that impedance can be used as a novel approach to monitor drug treatment and sensitivity of primary BCP-ALL cells in the presence of protective microenvironmental cells.

## Introduction

Normal B-cell lineage development occurs in bone marrow, where association with stromal cells regulates the early stages of progressive B-cell lineage maturation and selection [1–3]. Human B-cell precursor acute lymphoblastic leukemia (BCP-ALL) also originates in bone marrow. This is also a frequent location of primary relapse after conventional cytotoxic drug treatment. BCP-ALL cells are protected against chemotherapy by association with stromal cells. Such stromal cells act as a communication and instruction network, regulate local cytokines and chemokines levels, secrete extracellular matrix and overall create a microenvironment enhancing leukemia cell survival and proliferation [4–8].

We model this *ex vivo* by co-culture of human patient-derived xenograft (PDX) and primary BCP-ALL cells with OP9, murine bone marrow-derived stromal cells (mesenchymal stromal cells, MSC) that are widely used to support human stem cells and human T-cell development [9–12]. In this co-culture system, OP9 monolayers secrete the chemokine SDF1α, a known migration factor for human BCP-ALL [13, 14]. The leukemia cells migrate towards the monolayer, nestle underneath the stromal cells, and proliferate at that location. When BCP-ALL cell numbers sufficiently increase, they also migrate into the cell culture medium. When such co-cultures are used to follow drug sensitivity of BCP-ALL cells over time, viability or numbers of these floating cells are typically the targets that are counted. Therefore, although this system is able to represent some aspects of drug treatment, it does not score the effects of drugs on the BCP-ALL cells that are in the most proximal contact with the stroma, at the location where they are expected to be maximally protected [15]. To address this problem, we investigated the possibility of a novel approach to measure the effects of chemotherapy treatment on BCP-ALL cells in a co-culture set up using the xCELLigence system. That system, which continuously monitors cell proliferation while allowing the leukemia cells to remain undisturbed in their microenvironment, was evaluated and compared to traditional 2D methods.

## Materials and methods

### Cells and culture

The murine OP9 bone marrow stromal cell line (CRL-2749) and the human HS5 bone marrow stromal cell line (PCS-500-041) were obtained from the American Type Culture Collection (ATCC, Manassas, VA, USA). Cells were grown in OP9 medium (see below) and used at an early passage. In co-culture experiments, these cells were mitotically inactivated by irradiation for 16.7mins (74.31Gy) using a 137Cs irradiator. Alternatively, OP9 cells that had been plated and allowed to adhere overnight were treated with 10 μg/ml mitomycin C (Sigma cat M4287) for 3 hrs in complete medium, washed with media and used the next day for support of human BCP-ALL cells. For Transwell co-cultures, OP9 cells were plated in the bottom chamber and were separated from the leukemia cells by a 0.4 μm membrane filter (Corning, Cat# 3381).

US7 (also referred to as LAX7 in other studies), ICN13, BLQ1, and BLQ5 have been previously described [14, 16–18]. The STR genotype of US7 and ICN13 has been established. MUTZ5 and MHH-CALL-4 were purchased from the DSMZ (ACC 490 and ACC 337, respectively). US7, ICN13, BLQ1, and BLQ5 were cultured in 10 cm plates on a layer of OP9 stromal cells in MEM-α medium (Gibco, Thermo Fisher Scientific, Waltham, MA, USA, Cat# 12561–056) supplemented with 20% FBS (Atlanta Biologicals, Flowery Branch, GA, USA, Cat# D14047), 1% L-glutamine (Gibco, Thermo Fisher Scientific, Waltham, MA, USA, Cat# A29168-1) and 1% penicillin/streptomycin (Gibco Thermo Fisher Scientific, Waltham, MA, USA, Cat# 15140122). BM61, BM47 and BM13 were cryopreserved primary patient samples cultured as described for US7, ICN13, BLQ1 and BLQ5: in 10 cm plates on a layer of OP9 stromal cells in MEM-α medium supplemented with 20% FBS. BM61 (95% blasts: CD45+CD19-CD20+CD22+CD10+), BM47 (CD45+CD19-CD20-CD22-CD10+), and BM13 (95% blasts: CD45+CD19+ CD20+/-^(Dim to negative)^ CD22+CD10+) are relapsed primary BCP-ALL cells. BM61 and BM13 are Philadelphia chromosome (Ph) positive ALL cells. Ficoll-purified bone marrow mononuclear cells from BCP-ALL samples were cultured on irradiated OP9 stroma for approximately one week, as described previously for primary BCP-ALL cells first passaged in NSG mice [14, 18], before use in xCELLigence experiments. MUTZ5 and MHH-CALL-4 were cultured in T75 flasks in RPMI 1640 medium (Gibco, Thermo Fisher Scientific, Cat# 11875093) supplemented with 20% FBS, 1% L-glutamine, and 1% penicillin/streptomycin. OP9 stromal cells were mitotically inactivated by irradiation. Human leukemia cells were collected by gently pipetting the cell suspension without disturbing the OP9 stromal cells. OP9 cells were harvested using trypsin-EDTA (Thermo Fisher Scientific, Waltham, MA, USA, Cat #25300054) for 3 min in a humidified atmosphere with 5% CO_2_ at 37°C. All cells were cultured in a humidified atmosphere with 5% CO_2_ at 37°C. Cells were washed with 3 mL of PBS and centrifuged for at 230 x g for 5 min at 4°C during time of collection.

BLQ1, BLQ5, HS-5, ICN13, MHH-CALL4, MUTZ5, OP9 and US7 were tested and found to be mycoplasma-free. Mycoplasma testing was performed via the MycoAlert PLUS Mycoplasma Detection Kit (Lonza, Basel, Switzerland, Cat #LT07-705). These cells were also STR-genotyped and, if available, STR genotype was confirmed in comparison with publicly available profiles via University of Arizona Genetics Core.

### Ethics statement

All human specimen collection protocols were reviewed and approved by Children’s Hospital Los Angeles Institution Review Board (IRB) and Committee on Clinical Investigations (CCI). All methods were performed in accordance with the relevant guidelines and regulations. Collections were in compliance with ethical practices and IRB approvals. All specimens were de-identified/ anonymized before acquisition for research. BM61, BM47, and BM13 were collected as leftovers of samples that were initially collected for clinical diagnostic purposes and were discarded as medical waste when no longer needed for clinical purposes. US7, ICN13, BLQ1, and BLQ5 have been previously described [14, 16–18].

### Drugs

Tasigna (nilotinib, Novartis, Basel, Switzerland, NDC #0078–0951), ponatinib (MedChemExpress, Monmouth Junction, NJ, USA, Cat# HY-12047), vincristine (Hospira Worldwide Inc., Lake Forest, IL, USA, Cat# 61703030906), and VX-680 (Selleck Chemicals LLC, Houston, TX, USA, Cat# S1048) stock solutions were diluted in DMSO. Subsequent dilutions were made using MEM-α media. Ruxolitinib (INCB018424) was obtained from MedChemExpress (Monmouth Junction, NJ, USA, Cat # HY‐50856).

Concentrations of the drugs used were selected based on the IC_50_ values published in the literature, the drug manufacturer’s website, and PubChem. Nilotinib was tested at 1 nM– 100 nM (corresponding to a clinical dose of 0.00413–0.413 mg/m^2^ for a tissue culture well with 100 μL volume and 0.32 cm^2^ surface area), vincristine at 5 nM– 25 nM (0.0322–0.644 mg/m^2^), ponatinib at 0.2 nM– 12 nM (0.000832–0.0499 mg/m^2^), VX-680 [tozasertib] at 100 nM -1 μM (0.363–3.63 mg/m2).

### Cell growth and proliferation assay under drug treatment using the xCELLigence system

**Fig 1:**
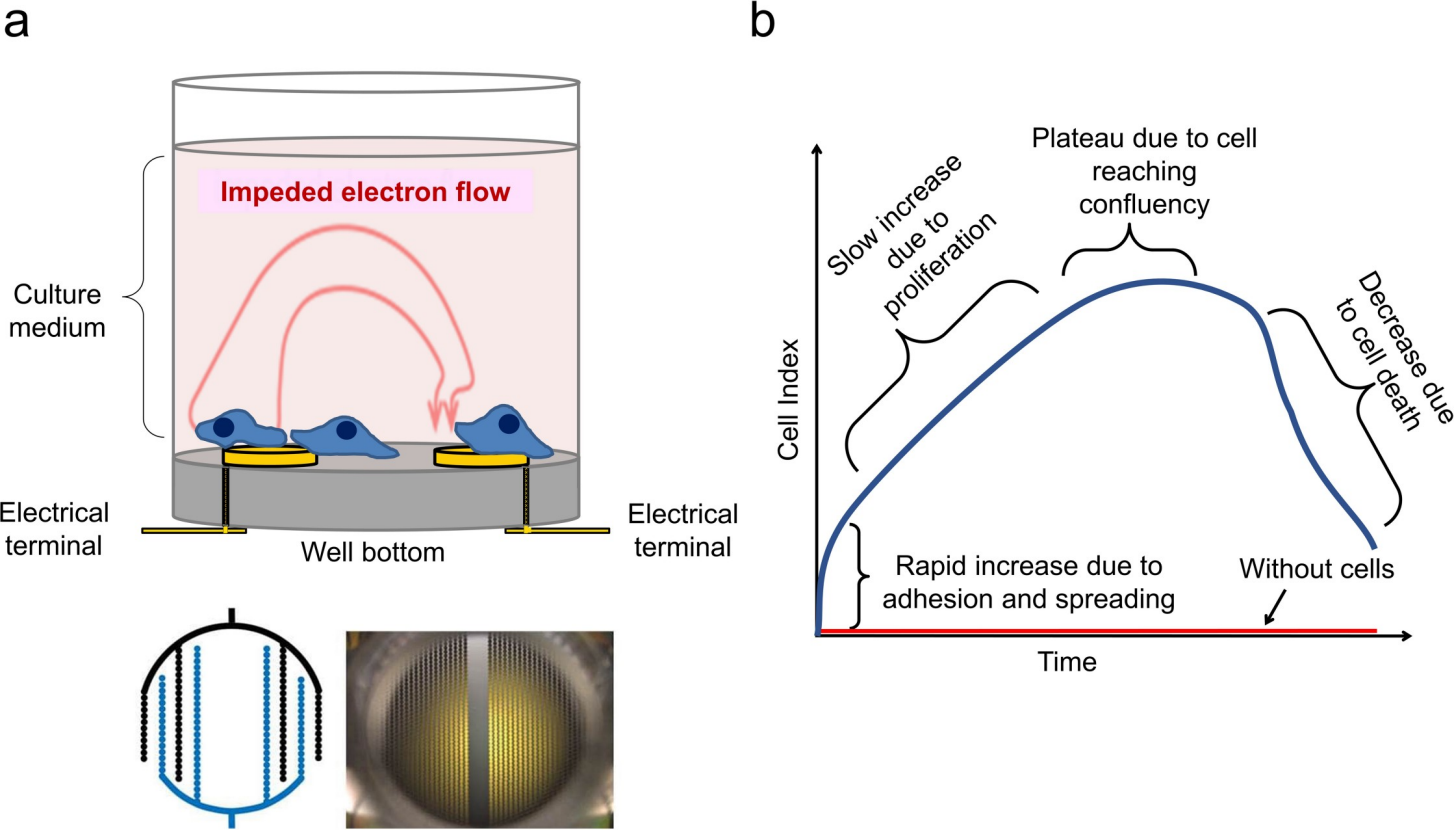
Overview of the xCELLigence impedance-based technology. (A) Multi-well plates have gold electrodes embedded on the bottom that measure the conductivity of the medium through a small current. Adherent cells cover the surface of the electrodes and impede the flow of the current. (B) Typical cell proliferation curve measured through the standardized impedance parameter (Cell Index) shows the different phases of initial cell adhesion, exponential growth, confluency and cell death. The Cell Index without cells is 0. This is based on [20].

The xCELLigence system (RTCA DP, Agilent Technologies, Santa Clara, CA, USA) was used to analyze BCP-ALL cells in co-culture with stromal cells. The system consisted of RTCA Resistor E-Plate 16 devices (Agilent Technologies, Santa Clara, CA, USA, Cat # 380601050) placed within the RTCA DP device housed in a humidified atmosphere with 5% CO_2_ at 37°C, and RTCA software (version 2.0) that was used to verify system conditions, sample monitoring and data analysis. The E-Plate 16 devices are cell culture plates that have gold microelectrodes embedded within each well (Fig 1A) allowing readings to be taken by the RTCA DP device. The wells of the E-Plate 16 devices connected to the xCELLigence system were coated with 10 μg/mL fibronectin (Thermo Fisher Scientific, Waltham, MA, USA, Cat #PHE0023) or 0.2% gelatin in DPBS (Sigma Aldrich, St. Louis, MO, USA, Cat #G1393-100ML) for 30 minutes at 37°C. Once the coating was removed, 50 μL of complete MEM-α media was added to each well and a background reading was recorded with the analyzer. OP9 cells (10,000 cells/well in 50 μL complete MEM-α media) were then seeded into each well and the plates were left at room temperature in a laminar flow hood for 30 minutes to allow the OP9 cells to settle. The plates were then placed back into the analyzer to allow the OP9 cells to adhere overnight to establish the feeder layer and to monitor them. Human leukemic cells were added to the wells with the established OP9 feeder layer (except where indicated in the Figure legend, 1 x 10^5^ cells/well in 100 μL complete MEM-α media) and left undisturbed for another 24 hr to allow migration underneath the feeder cells. Anticancer drugs (in 50 μL complete MEM-α media) were added after 24-hr co-culture and impedance was measured by RTCA software at 15 min intervals over a period of 7 days. The real-time cell index, representing changes in impedance from the initial background reading, was determined using the RTCA DP software, and a histogram representative of the cell index over 7 days was generated for each BCP-ALL/drug combination.

### Viability and apoptosis monitoring

Cell index values were derived from impedance readings of the E-Plate 16 devices. These plates were subsequently used for cell counting and apoptosis monitoring along with the wells from the 96-well tissue culture plates and HTS 96 well Transwell plates. Cells were harvested from plate wells by pipetting and wells were washed once with 200 μL PBS. 10 μL of sample was mixed with 10 μL of 0.4% Trypan blue (Gibco, Thermo Fisher Scientific, Waltham, MA, USA, Cat #15250061) by gently pipetting, and then 10 μL of the mix was loaded into a chamber of the hemocytometer. Manual counts were performed under a 10x objective according to standard methodologies. Annexin V assays were performed by resuspending the rest of the cells in 1x Annexin V Binding Buffer (BD Biosciences, Franklin Lakes, NJ, USA, Cat# 556454) and staining with Annexin V-FITC (BD Biosciences, Cat# 556419) and PE Mouse Anti-Human CD45 (BD Biosciences, Cat# 561866) for 15 min at room temperature in the dark. Stained cells were washed and suspended in binding buffer. The final cell suspension was stained with 7-AAD (Thermo Fisher, Cat# 00-6993-50) for 5 min at room temperature in the dark and was processed through a flow cytometer (BD FACS Canto II). Data was processed using the instrument’s software.

### Derivation of Cell Index (CI)

As described previously, a unit-less parameter termed cell index (CI) was derived to represent cell status, based on the measured relative change in electrical impedance detected by sensor electrodes from the initial background reading [19].

### % Displacement calculation, IC50 calculation, and statistical analysis

The % Displacement for a drug treated BCP-ALL sample was calculated according to the following formula:

$$\%\;Displacement=100\left[\frac{Cell\;Index\;(OP9\;Alone)-Cell\;Index\;(Treated\;Sample)}{Cell\;Index\;(OP9\;Alone)-Cell\;Index\;(OP9\;plus\;BCP-ALL)}\right]$$

IC50 was automatically calculated using RTCA Software. The Levenberg-Marquardt method is used to fit the concentration-Cell Index data, (C1, CI1), (C2, CI2), …, (Cn, CIn), to the 3-parameter or 4-parameter logistic dose response model to derive the IC50 values.

Prism 8 (GraphPad, San Diego, CA, USA) was used to calculate correlation between Cell Index (CI) versus apoptosis analysis parameters as well as live cell counts. Statistical significance was determined using one-way ANOVA, followed by Dunnett’s multiple comparison test using OP9 plus BCP-ALL samples as control. To compare values between different time points, plate and drug conditions, statistical significance was determined using two-way ANOVA, followed by Bonferroni posttests.

## Results

### BCP-ALL cells proliferation in co-culture can be measured through feeder cell displacement

The xCELLigence system works by measuring electron flow transmitted between gold microelectrodes, in the presence of an electrically conductive solution such as tissue culture medium (Fig 1A). Adhering cells disrupt the interaction between the electrodes and the solution, thus impeding electron flow. This resistance to alternating current is referred to as impedance, which can be described in arbitrary units called cell index (Fig 1B).

Since bone marrow stromal cells are typically adherent cells that have a relatively large footprint, while BCP-ALL cells are non-adherent, we asked if it would be possible to measure the migration of the BCP-ALL cells to the location underneath the MSC by displacement of these stromal cells off the substratum to which they attach. This effect is possibly measurable through the use of the xCELLigence system. This system uses tissue culture wells that have electrodes in the bottom, onto which adherent cells can be plated [21, 22]. Adhesion and reattachment of the stromal cells can then be monitored over time, without any label, as an increase over time in impedance (resistance) of an electric alternate current that flows through the medium to the electrodes (Fig 1).

**Fig 2:**
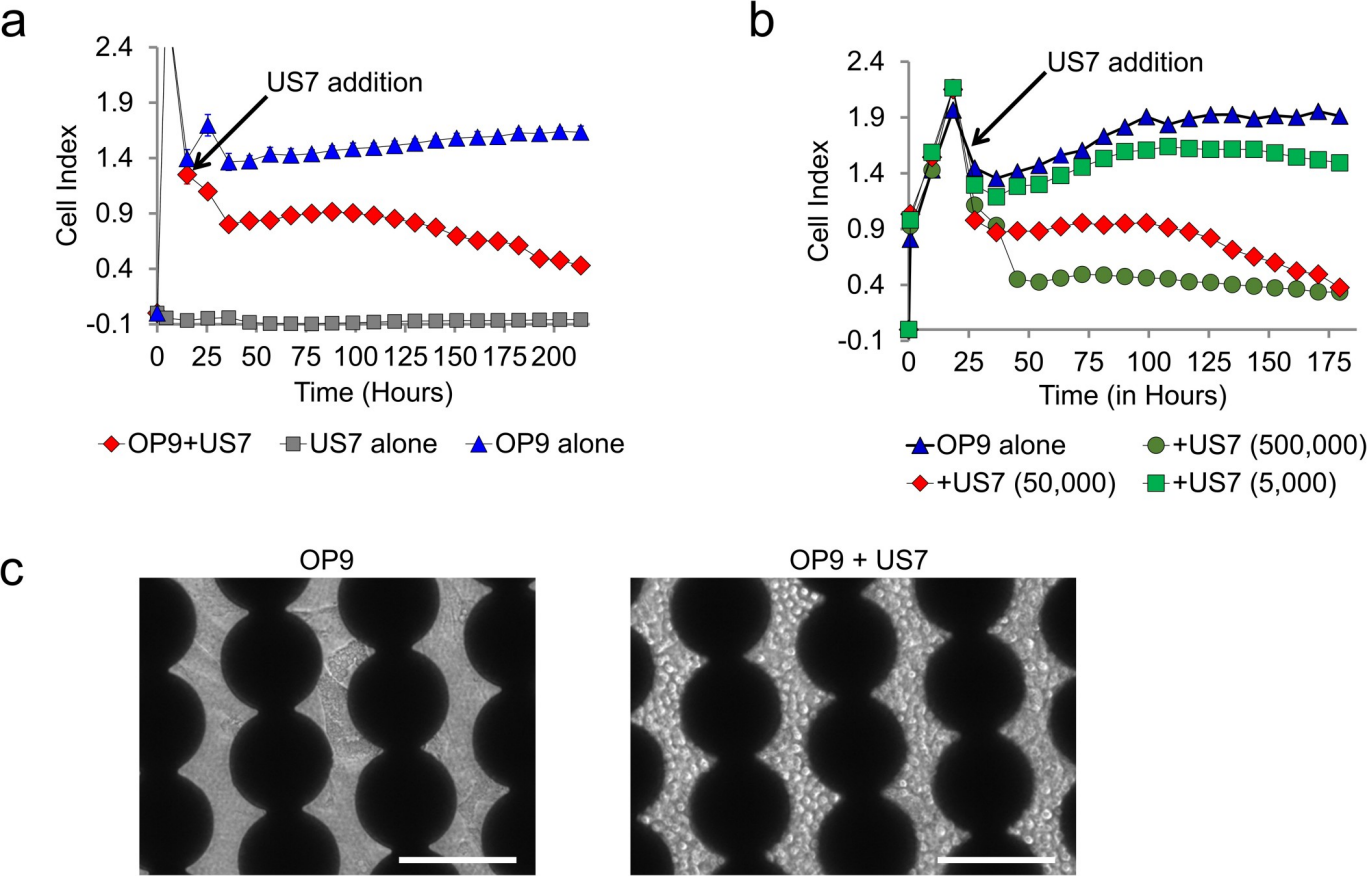
Impedance measurement of OP9 stromal cell adhesion and displacement by BCP-ALL cells. (A) Impedance was measured over 7 days in wells containing OP9 alone (10,000 cells/well), US7 alone (50,000 cells/well) and US7 plated on OP9 feeder cells as indicated (n = 2). When added to the well, OP9 stromal cells adhere to the electrodes and increase impedance (Cell Index) over time. PDX-derived BCP-ALL cells (US7) do not attach to the electrodes and do not affect the measured impedance; however, when co-cultured with OP9, they cause a reduction in impedance. (B) Impedance in co-culture of OP9 cells with different amounts of US7 cells as indicated below the figure (n = 2, triangle = no cells, diamond = 50,000 cells, square = 5,000 cells, circle = 500,000 cells). The reduction in impedance is proportional to the number of added US7 cells. (C) Bright field images of OP9 alone (10,000 cells/well) or OP9 and US7 BCP-ALL cells (50,000 cells/well) 7 days after leukemia cell addition. Dark areas are generated by electrodes blocking the light path in the inverted microscope. Cells are visible in the clear space between electrodes. Scale bar = 100 μm.

We plated mitotically inactivated OP9 BM MSC in this system. As shown in Fig 2A (blue line), these cells provided a high and constant impedance signal after they had attached to the electrodes. As expected, US7 human PDX-derived BCP-ALL cells, when added alone to a well, did not change the impedance (Fig 2A, grey line) as these cells remained floating in the medium and did not migrate to or adhere to the electrodes, even when these were coated with gelatin or fibronectin. Importantly, when US7 cells were added to wells that had been seeded with OP9 cells, there was a marked drop in impedance within the first 24–36 hours (Fig 2A, red line) as the leukemia cells migrated to and pushed underneath the OP9 cells, displacing their firm contact with the electrode.

Since the impedance progressively decreased with time, this suggested that the leukemia cells were increasing in numbers. To test this, we next added different amounts of US7 cells to a constant number of plated OP9 cells. As shown in Fig 2B, there was a clear dose-response, with lower numbers of US7 cells causing a smaller drop in impedance than higher amounts of cells, confirming that differences in impedance measures differences in BCP-ALL cells underneath the stromal cells. The concordance between reduction in impedance and proliferation of US7 cells was also supported by bright field images (Fig 2C).

**Fig 3:**
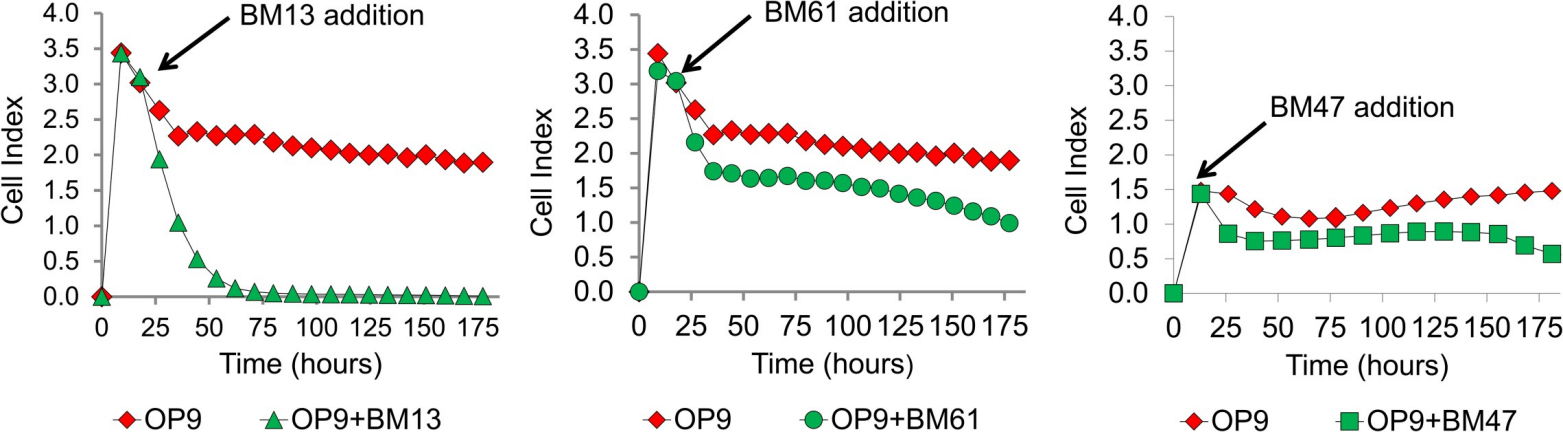
Different BCP-ALL cells exhibit unique patterns of impedance signal displacement. Primary (non-cultured) BCP-ALL samples Ph+ BM61, Ph+ BM13 and BM47 were plated (100,000 cells/well) one day after OP9 feeder cells (10,000 cells/well) and impedance was measured over more than 7 days (n = 2). BM13 and BM61 were run on the same plate, whereas BM47 was from a different experiment.

BCP-ALL cells derived from patients are expected to be unique because they contain different molecular lesions and can be stratified into different risk categories depending on identified driver mutations. One poor-prognosis subtype described as Philadelphia positive (PH+) contains the fusion of two genes, resulting in a deregulated tyrosine kinase called BCR/ABL1 [23]. As these molecular lesions could affect their migration and adhesion towards the OP9 stromal cells as well as their proliferation rates, we compared growth of primary BCP-ALL cells from three different relapsed patients. All tested BCP-ALL cells reduced the OP9 impedance, but with kinetics and profile that were unique for each sample (Fig 3), likely reflecting differences in their growth properties but also in interaction with the stromal environment. For example, Ph+ BM13 cells generated a stronger effect, suggesting these cells proliferated more robustly. After the initial displacement of OP9, Ph+ BM61 gradually reduced OP9-associated impedance, indicating a relatively slow proliferation. The growth of BM47 only became evident after day 6 of culture after the initial displacement. Thus, further studies with large sample numbers could be done to determine if a correlation exists between impedance, the interaction with the stromal environment of these cells and the driver oncogenes expressed by them.

### Selective drug cytotoxicity can be measured through co-culture displacement/impedance analysis

We next investigated if the effect of cytotoxic drug treatment on BCP-ALL cells can be monitored using this indirect OP9 displacement approach. US7 cells were isolated from a patient at diagnosis, and are sensitive to treatment with vincristine, a component of standard, first-line chemotherapy. Vincristine is an inhibitor of microtubule polymerization and is cytotoxic because it inhibits cell division. Therefore, it should not have an effect on the OP9 cells, which have been mitotically inactivated through irradiation. As shown in S1 Fig, we verified lack of effect on these BM-MSC cells through impedance monitoring.

**Fig 4:**
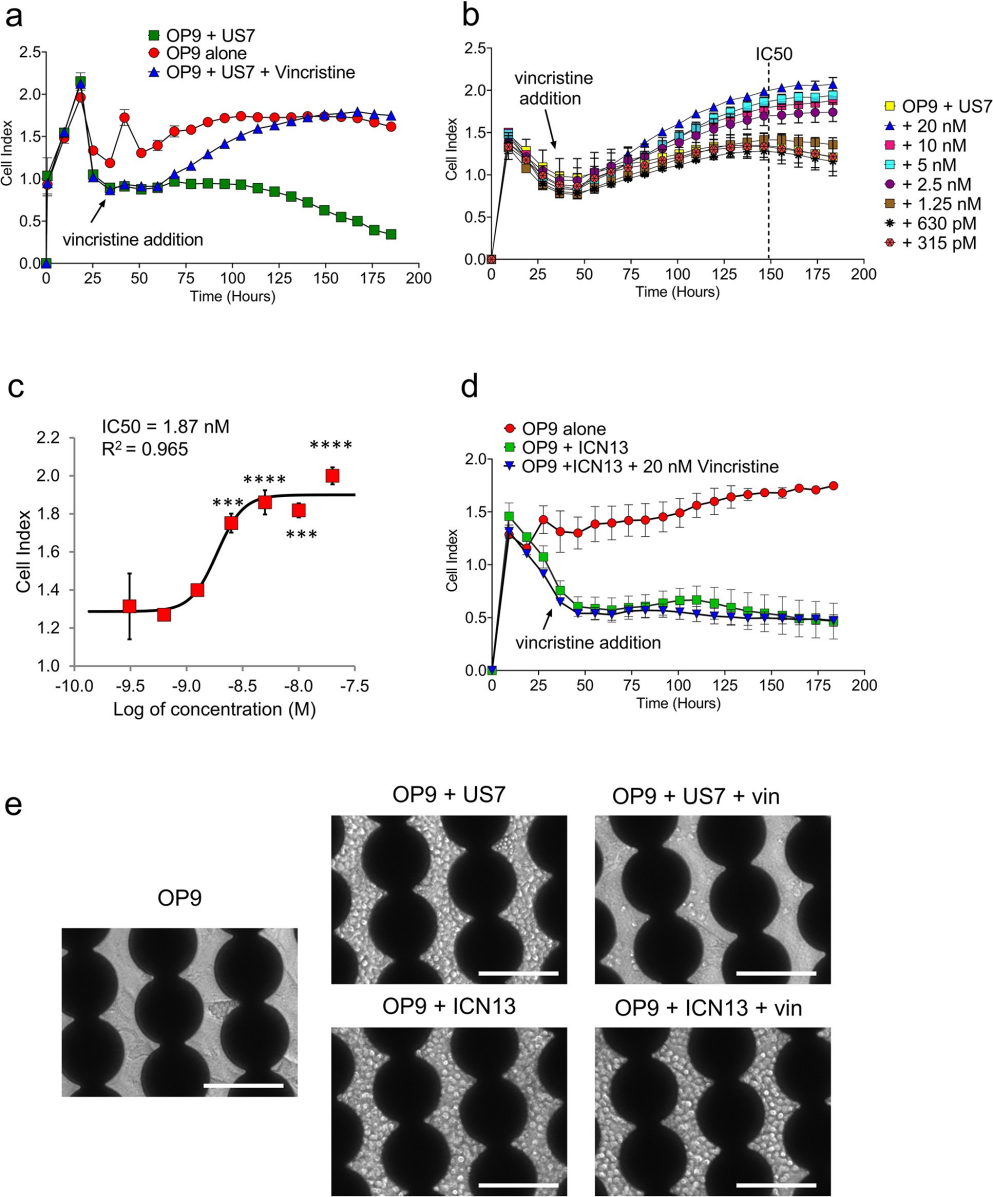
OP9 displacement can be used for monitoring drug cytotoxicity on BCP-ALL cells. (A) US7 cells treated with 100 nM vincristine as indicated (n = 2). (B) Cell Index profile of dose-response to vincristine treatment of OP9+US7. Dotted line indicates the time point used for calculating the IC50 (n = 3 except 1.25nm and 315pm n = 2). (C) Dose-response curve and IC50 calculated from the Cell Index profile in panel B. The IC50 value for vincristine treatment of US7 was 1.87 nM. Statistical analysis was done using one-way ANOVA followed by Dunnett’s multiple comparison test. Error bars represent standard deviation. p values relative to control: ***<0.001; ****<0.0001 (D) ICN13 cells treated with 20 nM vincristine (n = 2). (E) Images of cells indicated in the figure inside the 16-well plate devices, captured after 7 days of treatment with 10 nM (US7) or 20 nM (ICN13) vincristine. Scale bar = 100 μm.

We next treated the US7 BCP-ALL cell co-culture with vincristine. As shown in Fig 4A, whereas US7 in co-culture with OP9, as expected, reduced impedance caused by the OP9 cell adherence, treatment with chemotherapy had a marked effect: within about 48 hours after addition of vincristine the impedance began to increase, and upon continued chemotherapy reached the levels seen with OP9 alone cells. This indicates that the OP9 cells re-adhered firmly to the electrode after the leukemia cells had been eradicated with this drug.

To determine if this could be used to determine IC_50_ values, we treated US7 cells with a range of vincristine concentrations varying from 315 pM to 20 nM. Fig 4B shows that changes in impedance correlated with the dose of vincristine used to treat these BCP-ALL cells. After 48 hours, the Cell Index (CI) value increased in a dose-dependent fashion, compared to the US7 cells that did not receive any vincristine treatment. Using the CI value at 150 hours from the beginning of the experiment, we were able to calculate an IC_50_ for US7 cells at 1.8 nM (Fig 4C). We also treated ICN13 cells, which are drug resistant in our standard OP9 co-culture (S2 Fig), with vincristine in the xCELLigence system (Fig 4D). In concordance with their lack of reaction to this cytotoxic drug, impedance of ICN13 co-cultures with and without vincristine treatments were identical (Fig 4D) and no IC_50_ could be determined due to lack of sensitivity to all tested concentrations (S3 Fig). Imaging of the US7 cells and ICN13 cells (Fig 4E) supported the concept that US7 co-cultures treated with vincristine had a marked decrease in BCP-ALL cell numbers, because the OP9 feeder layer became more visible, as compared to the ICN13 co-culture, which did not have reduced BCP-ALL cell number under vincristine treatment.

**Fig 5:**
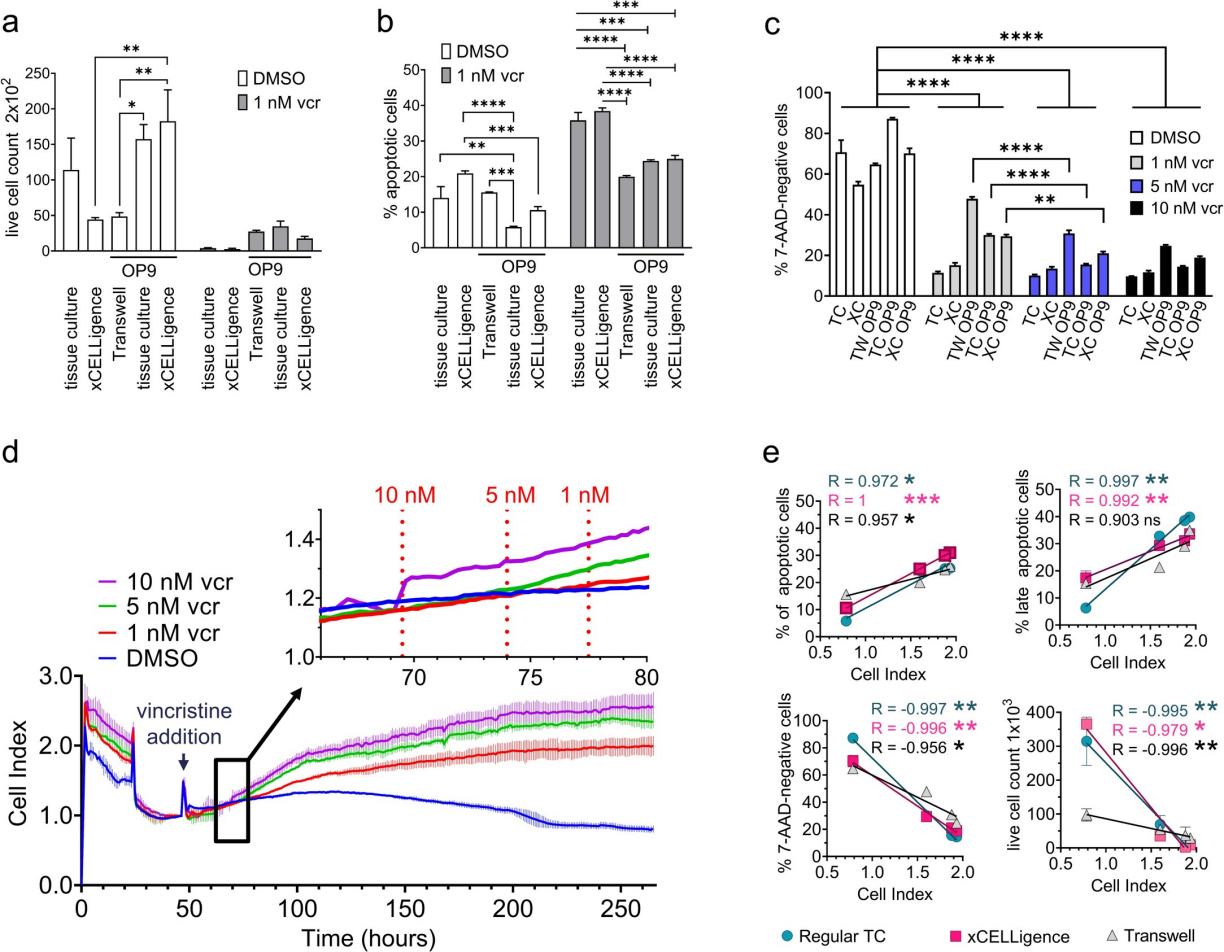
Comparison of end-point tissue culture with OP9 displacement. US7 cells were plated on 96-well tissue culture plates (TC = tissue culture), HTS 96 well Transwell plates (TW = transwell), and E-Plate 16 devices (XC = xCELLigence) as indicted in the figure (all coated with fibronectin) and exposed to either DMSO or 1 nM vincristine (vcr). On day 9, cells were harvested and examined for (A) live cell numbers using Trypan blue exclusion (B) apoptotic cells by FACS using 7-AAD and Annexin V (C) living cell percentages based on FACS and negativity for 7-AAD. Statistical analysis was done using two-way ANOVA, followed by Bonferroni posttests. Error bars represent standard deviation. p values: *<0.05, **<0.01; ***<0.001; ****<0.0001. (D) xCELLigence continuous monitoring of parallel cultures under the indicated drug treatments (n = 3). Dotted lines in inset indicate time points of early differentiation visible between the controls and treatment wells. (E) Correlation of cell index values with conventional cell viability measurements. Error bars represent standard deviation. p values of correlation coefficient: *<0.05, **<0.01; ***<0.001.

We next compared this system with a standard end-point culture after 9 days of treatment with vincristine. As shown in Fig 5A (DMSO), US7 cells plated only on fibronectin-coated xCELLigence plates without stromal support, and US7 cells not directly contacting OP9 stromal cells by separation through a transwell membrane had lower live cell numbers than BCP-ALL cells growing in direct contact with stroma. The percentage of spontaneous apoptosis was also lowest in direct co-culture (Fig 5B, DMSO). OP9 stromal support furthermore protected the leukemia cells against 1 nM vincristine (Fig 5B, grey bars) compared to cultures without OP9. Compared to the DMSO-treated controls, US7 cells exposed to 1, 5 and 10 nM vincristine showed clearly decreased live cell numbers (Fig 5C) but the distinction between 5 and 10 nM was small. Under continuous monitoring (Fig 5D) there was an early differentiation visible between the controls and 1, 5 and 10 nM between 21 h and 30 h post drug addition which was maintained till the end of the observation period on day 9. A comparison of the cell index values versus terminal counts confirmed the correlation between cell index versus apoptosis, live cells, and cell counts on day 9 (Fig 5E).

### Coculture monitoring can potentially reveal complex mechanisms of drug resistance

Ph-chromosome positive ALLs can be treated with targeted tyrosine kinase inhibitors (TKI). However, drug resistance frequently emerges with such cells harboring point mutations in the ATP binding site of *ABL1* which prevent binding of the inhibitor. To address if xCELLigence analysis could be used to detect this, we tested two Ph-positive ALL cell lines, BLQ1 and BLQ5, which both contain a T315I mutation [24] that makes them insensitive to the second generation TKI nilotinib. However, the third generation TKI ponatinib was designed to be able to inhibit the T315I mutant [25, 26], and the Aurora kinase inhibitor VX-680 also has “off target” inhibition of the *BCR/ABL1* T315I mutant [27, 28]. All drugs except VX-680 are FDA-approved. None had an effect on the OP9 cells (S4 Fig).

**Fig 6:**
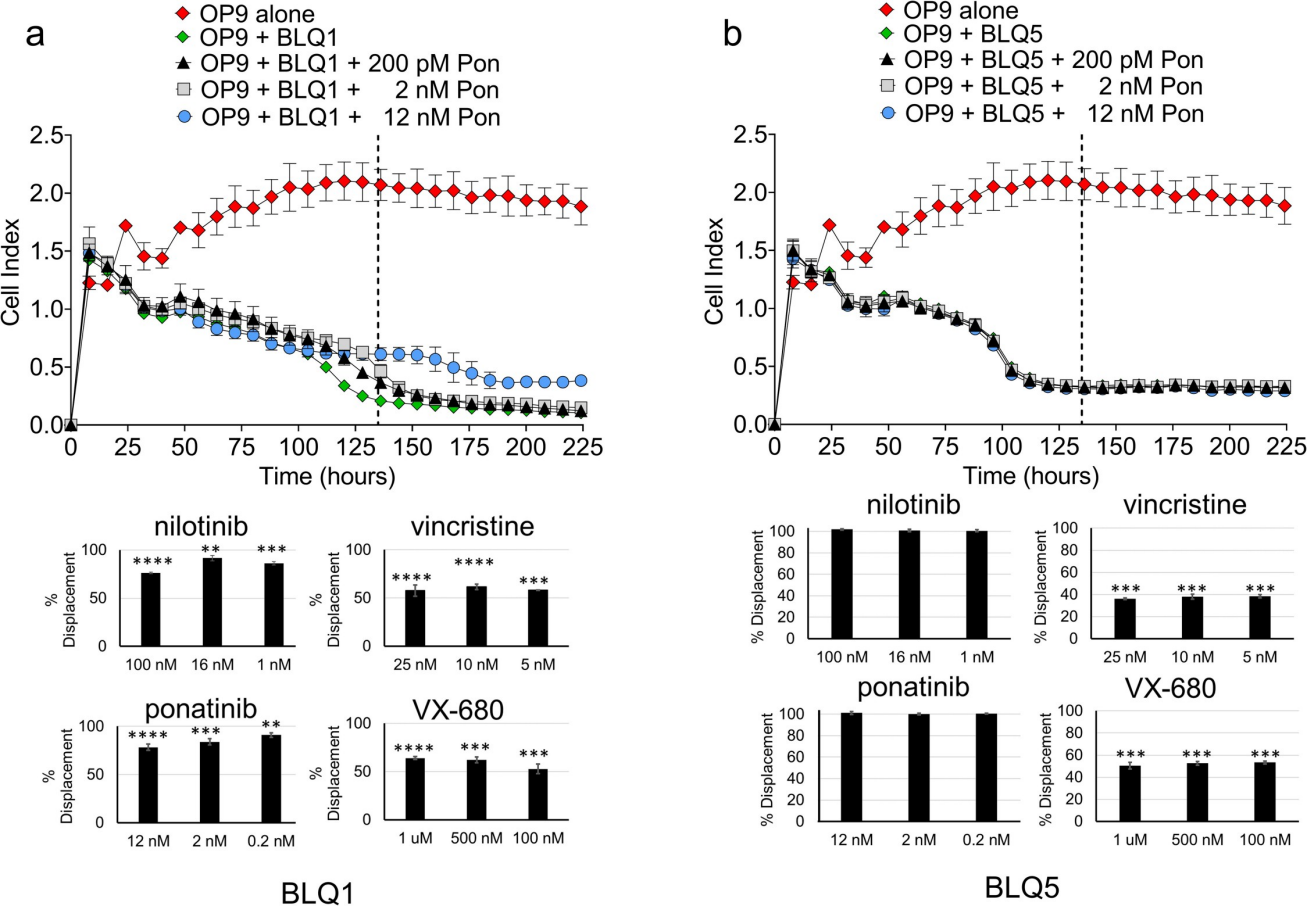
Different drug treatments generate unique patterns of impedance signal displacement in BCP-ALL cell co-cultures. Ph-positive BCP-ALL cells include BLQ1 (A) and BLQ5 (B) cells. *Top graphs*: different concentrations of ponatinib (pon). Dotted lines show the time point used for the % displacement calculation (also see S3 Fig). *Bottom graphs*: Percentage impedance signal displacement from treatment of BLQ1 and BLQ5 with the indicated concentrations of four drugs at the time points indicated in S3 Fig. Statistical significance was determined using one-way ANOVA, followed by followed by Dunnett’s multiple comparison test. Error bars represent standard deviation (n = 3). p values relative to control: *<0.05; **<0.01; ***<0.001; ****<0.0001.

BLQ1 had a modest response to 12 nM ponatinib and 100 nM nilotinib (Fig 6A, S5 Fig left panels), whereas impedance in the BLQ5 co-culture was totally unaffected (Fig 6B, S5 Fig right panels). Interestingly, proliferation of both BLQ1 and BLQ5 were affected by VX-680, possibly because of its additional activity as Aurora kinase inhibitor (S5 Fig). The inhibition of proliferation of BLQ5 was maintained over the course of the measurements, whereas the cytostatic effect on BLQ1 waned over time, suggesting that inhibition of proliferation was gradually overcome (S5 Fig). We also treated both BCP-ALL cells with vincristine as cytotoxic therapy. BLQ1, although somewhat responsive, appeared to start re-proliferation similar to its response to VX-680 whereas BLQ5 cells were more sensitive, and impedance after 225 hours approached that of OP9 cells alone, indicating significant BCP-ALL cell killing (S5 Fig). It should be noted that such differences in the response kinetics would be difficult to identify with end-point assays because such differences first became apparent at different time points. The responses of BLQ1 and BLQ5 to 16 nM nilotinib would have looked the same if the analysis had been performed 66 hours (no displacement) or 150 hours (full displacement) after treatment (S5 Fig). Thus, the detailed kinetics of the xCELLigence technology allows clear differentiation in the response.

**Fig 7:**
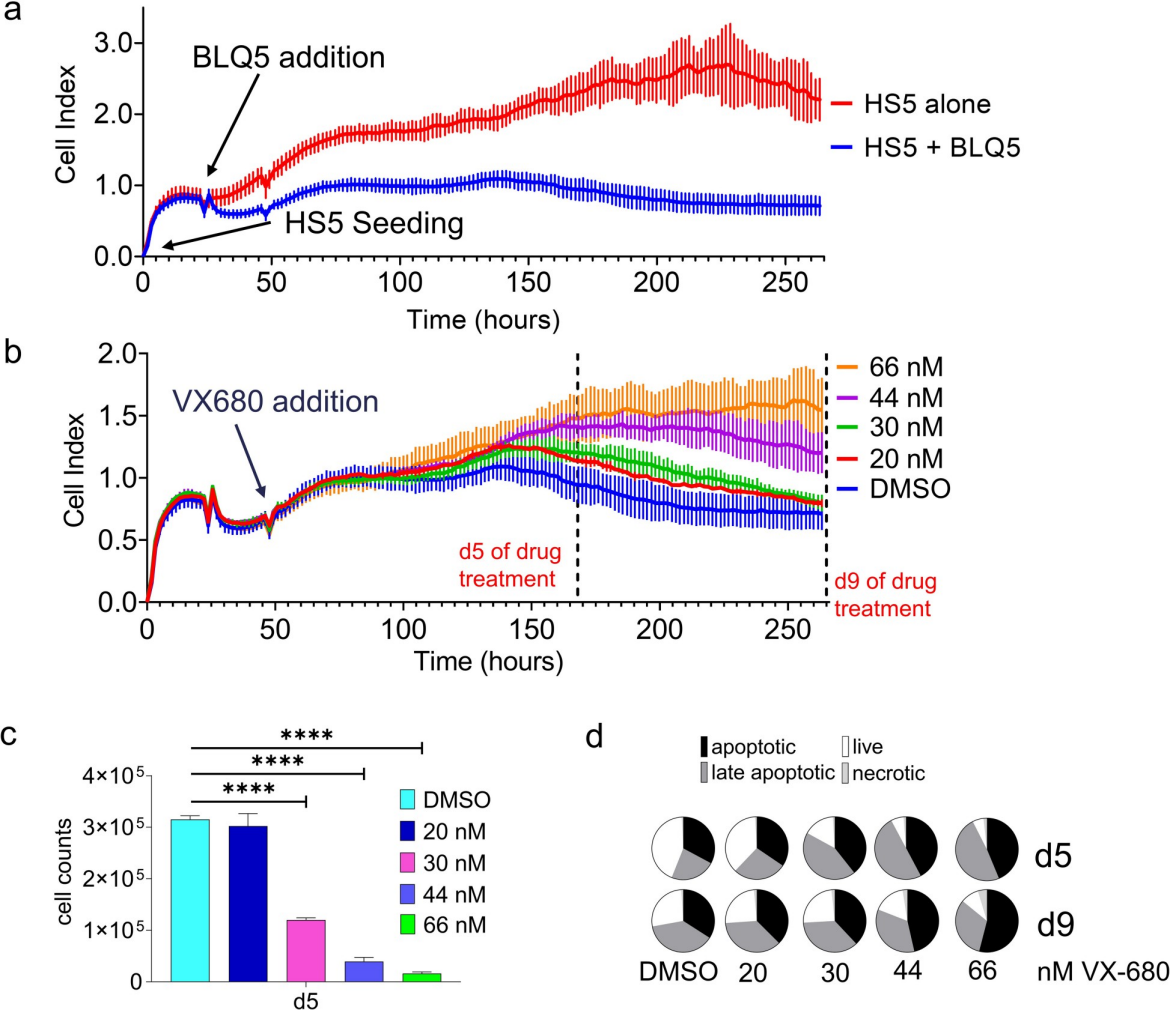
Treatment of BLQ5 co-cultured on human MSC with VX-680. (A) BLQ5 cells were plated on HS5 stromal cells, resulting in a reduction of impedance (n = 3). (B) Treatment of BLQ5 cells with different concentrations of VX-680 as indicated (n = 3). Dotted lines show the d5 and d9 time points at which cells were harvested for (C) live cell numbers determined by Trypan blue exclusion. Statistical significance was determined using one-way ANOVA, followed by followed by Dunnett’s multiple comparison test. Error bars represent standard deviation. p values relative to DMSO control: ****<0.0001. or (D) Percentage apoptotic cells characterized by FACS using 7-AAD and Annexin V.

Although OP9 cells are excellent at supporting BCP-ALL cell proliferation and survival, some applications may need to utilize human MSC stromal support. We therefore tested the human MSC cell line HS-5, which has a similar pattern of gene expression to primary bone marrow MSC [29] and is widely used to support survival of malignant human hematopoietic cells [30, 31]. As shown in Fig 7A, mitotically inactivated HS-5 cells plated alone have attached to the electrodes, causing an increase in cell index values over time. BLQ5 cells added to these plates displaced these stromal cells as they proliferated. We then treated the cultures with VX-680 and monitored the effect of drug treatment on the cell index over time. As shown in Fig 7B, effects of the drug were measurable as an increase in the cell index after about 2 days post VX-680 addition and on d5 there was a clear difference between control DMSO and 20 nM VX-680. Examination of parallel tests of BLQ5 on HS-5 in tissue culture plates using Trypan blue live cell counting similarly documented effects of the drug but did not clearly show an effect at the lowest drug concentration (Fig 7C). We also evaluated the cells in end-point cultures for percentages of live and apoptotic cells using a FACS-based assay (Fig 7D and S1 Table). These experiments show that specific human MSC can substitute for OP9 cells to provide chemoprotection. However, seeing that OP9 cells consistently supported higher BCP-ALL cell numbers and viability in long-term culture (S6 Fig), subsequent experiments made use of OP9.

**Fig 8:**
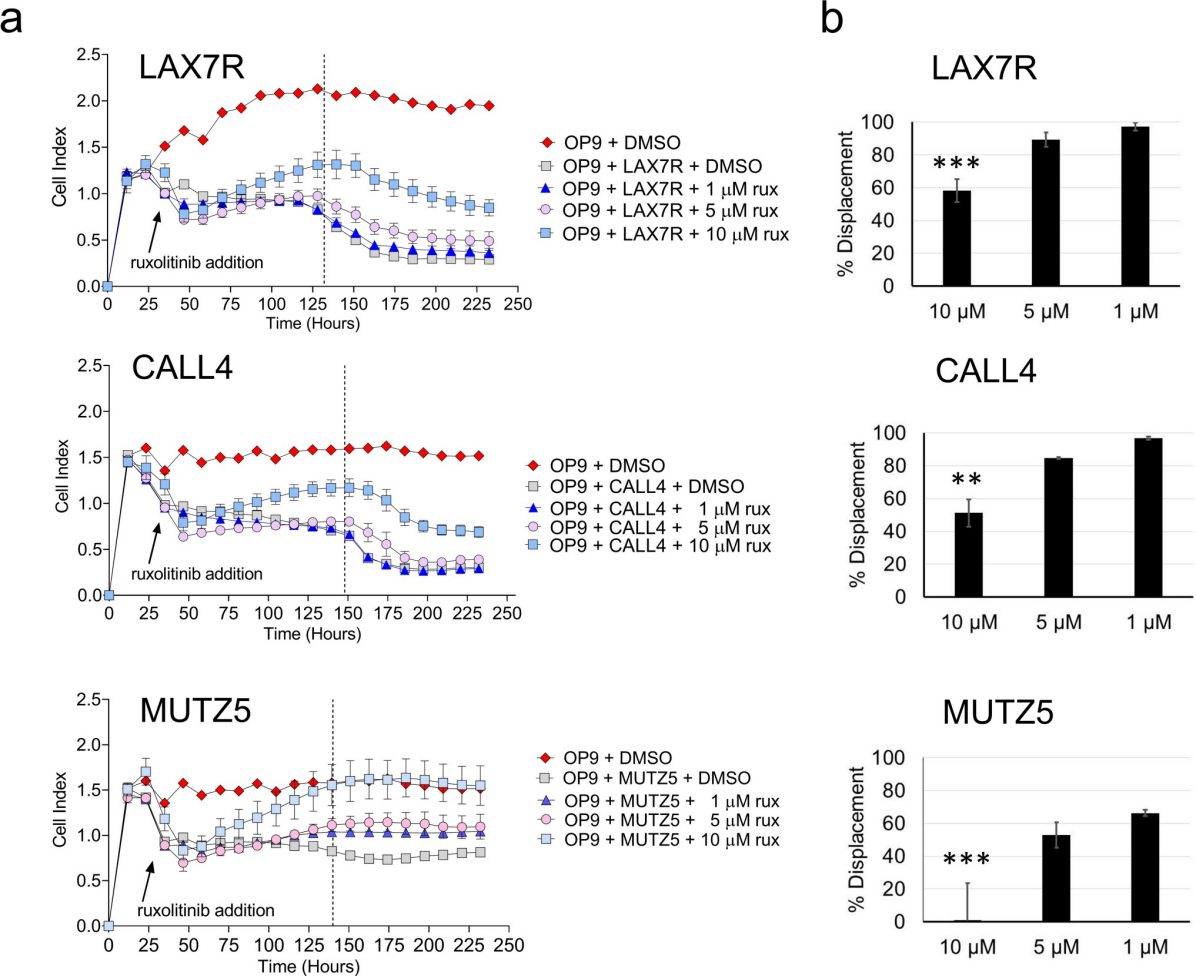
OP9 displacement can be utilized to test new drugs for BCP-ALL treatments. (A) Impedance profile of three BCP-ALL cell lines treated with the JAK2 inhibitor ruxolitinib (rux) (n = 3). Dotted lines show the time points used for the calculation of % displacement in panel B (also see S4 Fig) (B) Percentage impedance signal displacement from treatments with the indicated drug concentrations (n = 3). Statistical analysis was done using one-way ANOVA followed by Dunnett’s multiple comparison test to compare treatment samples versus the OP9+ALL+DMSO control wells. Error bars represent standard deviation. p values relative to control: *<0.05; **<0.01; ***<0.001; ****<0.0001.

We next determined if we could measure responses to the Janus kinase 2 (JAK2) inhibitor ruxolitinib, which is in clinical trials for treatment of a subclass of Ph-like ALLs (NCT02723994). We first tested LAX7R, which expresses constitutively active Ras (K-RasG12C mutation, see [24]). As shown in Fig 8A, after around t = 100 hours, the obvious displacement of the OP9 cells by LAX7R (compare red and grey lines) had clearly been reduced by treatment with 10 μM ruxolitinib (light blue line), which had little effect on OP9 cells alone at this time point (S7 Fig).

We next tested MUTZ5 and MHH-CALL-4 as two BCP-ALL cell lines that should be sensitive to this drug: both cell lines overexpress *CRLF2* via an *IGH@-CRLF2* translocation. MUTZ5 and MHH-CALL-4 additionally contain JAK2 R683G and JAK2 I682F mutations, respectively [32]. As shown in Fig 8 (grey lines), we found that OP9-associated impedance was clearly reduced due to the growth of MHH-CALL-4, indicating displacement of the stromal cells. As mentioned, importantly, ruxolitnib only had a transient effect on the OP9 cells (S7 Fig) whereas a clear dose-response was observed with both the MHH-CALL-4 and MUTZ5 cells (Fig 8A and 8B).

## Discussion

The current study was taken to explore the possibility of applying the xCELLigence impedance-based system to monitor chemotherapy treatment on BCP-ALL cells while they are in co-culture with BM stromal cells. When kept in the presence of stromal cells, but without direct cell to cell contact, leukemic cells can undergo spontaneous apoptosis, with viability significantly dropping over time [33–35] (see also Fig 5B). With a direct contact co-culture system, viability bias due to spontaneous apoptosis is reduced and long-term monitoring of the cells in their protective physiological environment obtained without the necessity of adding external factors such as cytokines that have only partially been identified.

The system studied here offers a middle ground between 2D and 3D culture. While 3D cultures can provide environmental “niches” that allow proper cell-cell interactions and mimic natural structures of tissue, they often result in low reproducibility and require expert handling [36]. Thus the current system can be viewed as a 2D culture system with a “set-and-forget” protocol feature. In contrast, most standard *in vitro* assessments involve the measurement for viable cells at single time points, generally 72 h after drug addition, [37] which may be too short to detect responses that, in patients, may take longer to become evident. With the xCELLigence system, the leukemic cells can be continually monitored for at least 7 days, while in direct contact with BM stromal cells, providing longer kinetic data in a system more comparable to the *in vivo* microenvironment. In addition, the xCELLigence system allows label-free monitoring, reducing manipulation and alteration of cell physiology, while the instrument software allows for scheduled time interval measurements without the necessity of manual computation. Finally, data from various time points are acquired from a single well, resulting in a reduction of experimental error. There is also an economical advantage in comparison to traditional methods that require extensive hands-on time involvement and reagents to generate equivalent data measurements.

Here, the system is used to indirectly monitor cell proliferation rates in a non-invasive manner because cells are not counted by physically removing them from the cultures, but by observing the rate of change they cause in impedance shifts: a faster drop in impedance would imply an increased rate of cell proliferation, with the slope used to measure the speed of cell division. This was evident when we compared Ph+ BM61 to Ph+ BM13, with the latter growing more rapidly.

The real-time and continuous monitoring feature of the system allow the possibility of assaying the type of effect of drug treatment: cytostatic and cytotoxic drug activity can be distinguished from each other by observing the pattern of impedance displacement over a detailed kinetic. With cytostatic drugs such as the tyrosine kinase inhibitors used here, cell proliferation is inhibited. Although the leukemia cells are no longer dividing, BCP-ALL cells that had displaced the OP9 at the beginning of the experiment are still attached to the electrodes, maintaining a lower plateau CI value in such cultures compared to the plateau value for OP9 cells alone. In contrast, in the case of a cytotoxic drug such as vincristine, apoptosis of BCP-ALL cells occurred. As they died, the OP9 or human HS5 stromal were able to reattach to the electrodes, increasing the impedance and resulting in a plateau similar to that of stromal cells alone. The xCELLigence system could possibly also flag emerging long-term resistance, when curves would display a pattern in which initially a peak of impedance appears, indicating cell death, followed by a drop, indicating recovery of BCP-ALL cells and re-initiation of growth even under continued drug treatment.

It is also possible to empirically detect subtle and less subtle differences between individual BCP-ALLs as to how they react to specific drugs, which allows a quantitative assessment with respect to ‘drug resistance’ and could be helpful in determining chemotherapy approaches to patient treatment. For example, complete resistance at a certain drug dose was illustrated by the lack of increase in impedance after drug treatment in US7, while with ICN13, cell proliferation continued, resulting in the CI value dropping until a plateau was reached when the BCP-ALL cells displaced the maximum possible OP9 cells from the electrodes.

Using BLQ1 and BLQ5, we were also able to detect unexpected differences, as determined by divergent curve patterns in a side-by-side comparison. We found that BLQ5 was resistant to ponatinib whereas BLQ1 exhibited some sensitivity. This could not have been predicted based on sequencing of the ATP binding site of the oncogene that drives these leukemias: both BLQ1 and BLQ5 were isolated from patients who had relapsed and both contain the Abl T315I point mutation but lack other additional mutations in the ATP binding site that could explain differential resistance to ponatinib [38, 39]. Interestingly, BLQ1 expresses *BCR/ABL1* p210 whereas BLQ5 expresses *BCR/ABL1* p190 as driver oncogene [24]. In mouse studies, the p190 form was found to produce a more aggressive B-cell lineage leukemia [40] and the two proteins also differ in their interactome [41]. While a similar response between BLQ1 and BLQ5 to the standard chemotherapeutic vincristine was expected due to their driver mutations, the degree of differential sensitivity to the treatment could not have been predicted.

In addition, we tested sensitivity of two cell lines representative of Ph-like ALL, both carrying *CRLF2* translocations but with different activating JAK2 mutations, for their sensitivity to the JAK2 kinase inhibitor ruxolitinib (Jakafi^®^). Here too the system was able to tease out subtle differences in drug sensitivity, with 10 μM ruxolitinib having a relatively potent effect on MUTZ5, whereas MMH-CALL-4 had a much less deep or durable response to the same dose. In our experiments, the treatment of OP9 cells with ruxolitinib caused changes in morphology measured by changes in impedance, although this was transient and did not preclude its testing in this setting. Indeed, ruxolitinib may also have effects on cells in the bone marrow other than the intended target BCP-ALL cells. For example, AlMuraikhi et al [42] reported that human BM MSC treated with 3 μM ruxolitinib had differential expression of more than 1500 genes compared to vehicle-treated controls. However, because leukemia cells treated with the inhibitor are presumed to be dependent on activation of the Jak/STAT pathway, whereas normal cells are not, ruxolitinib treatment does not appear to have consequences for non-leukemia cell survival: Jakafi^®^ is clinically approved for treatment of myelofibrosis in adults as well as for acute graft‐versus‐host disease.

It is possible that other drugs, such as F-actin disrupting compounds, could not be tested in the xCELLigence system because they would cause detachment of the OP9 stromal cells from the plate. Thus applications of this system are limited to drugs that do not cause permanent cellular detachment. However, if it is the experimental intent to test drugs that could be used for treatment of human BCP-ALL, this finding could also be early evidence of systemic toxicity. We conclude that because some primary BCP-ALL samples, and in particular those from relapsed patients, are able to grow in co-culture with OP9, the co-culture system described here could be a valuable assay platform to empirically assess the sensitivity of BCP-ALL cells to different second-line chemotherapy treatments.

## Supporting information

S1 Fig Vincristine has no effect on irradiated OP9 feeder cells. Mitotically inactivated OP9 cells were plated at a concentration of 10,000 cells per well and treated with different concentrations of vincristine as indicated (n = 3 except 100nm n = 2).
(TIF)

S2 Fig ICN13 are resistant to vincristine compared to US7 cells. US7 and ICN13 BCP-ALL cells (5x10^4^) plated on irradiated OP9 stroma were treated with vincristine in the same experiment. Viability of cells was determined at 535–612 nm after a 5 hr incubation with 10% v/v Alamar blue.
(TIF)

S3 Fig ICN13 and varying concentrations of vincristine: Impedance curves showing inability to calculate IC50. The dotted line indicates the time point used in the attempt to calculate IC50 (n = 3).
(TIF)

S4 Fig Lack of effect of targeted small molecule inhibitors on impedance of mitotically inactivated OP9 stromal cell layer. OP9 cells were mitotically inactivated, then plated at a concentration of 1x10^4^ cells and treated with the indicated concentrations of ponatinib, nilotinib, vincristine, and VX-680 as shown in the figure (n = 3). BLQ1 plated on OP9 cells is included as a reference plot.
(TIF)

S5 Fig Treatment of BLQ1 and BLQ5, two Ph-positive BCP-ALLs containing Bcr/Abl1 T315I mutations, with ponatinib, nilotinib, vincristine, and VX-680 as indicated. The percentage of displacement was determined approximately 125–150 hours after plating (n = 3). At this time point, the rate of change of the CI value was at its highest. Dotted lines show the time point used for measuring the % displacement in Fig 6A, 6B, bottom graphs. * identifies the time of BCP-ALL cell addition, whereas the arrowhead indicates the moment of addition of the drug.
(TIF)

S6 Fig Comparison of human BCP-ALL cell growth on human (h) MSC and OP9. Human leukemia cells as indicated were grown on mitotically inactivated stromal cells and standard tissue culture plates as indicated (n = 2). Viable cell counts were determined by Trypan Blue exclusion. PC and AT, two different primary MSC sources.
(TIF)

S7 Fig Ruxolitinib transiently changes the morphology of OP9. Mitotically inactivated OP9 cells were plated at a concentration of 10,000 cells per well and treated with different concentrations of ruxolitinib as indicated (n = 1). Note that the drug treatment only affected the CI/morphology of the cells up to 100 hours from the beginning of the experiment.
(TIF)

S1 Table Percentage apoptotic cells characterized by FACS using 7-AAD and Annexin V. (XLSX)
