# Supplementary figures and images for: Analysis of acute lymphoblastic leukemia drug sensitivity by changes in impedance via stromal cell adherence
Source: PLoS One. 2021 Sep 30;16(9):e0258140. doi: 10.1371/journal.pone.0258140 (PMC8483355; doi:10.1371/journal.pone.0258140)

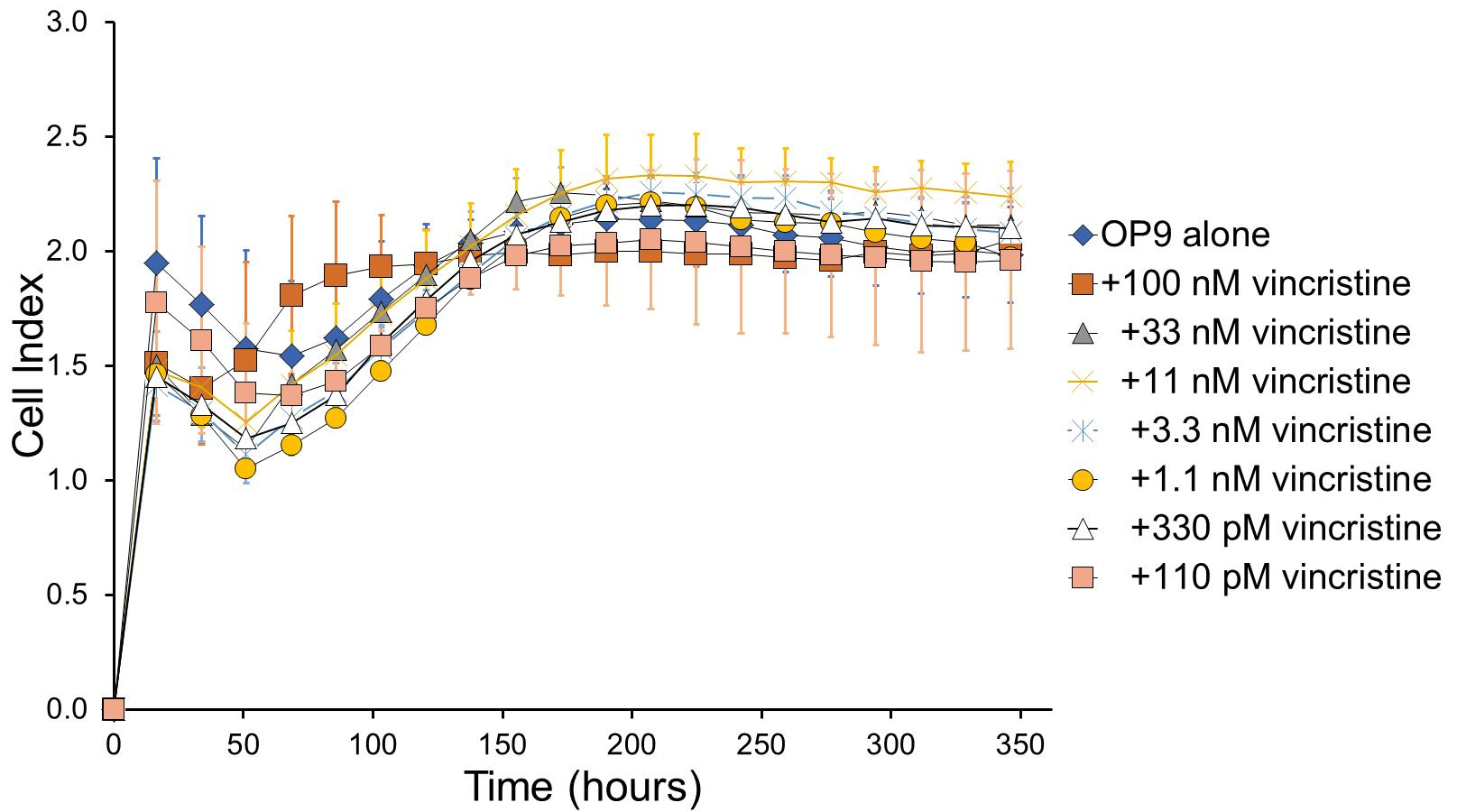

Supplement: S1 Fig — Mitotically inactivated OP9 cells were plated at a concentration of 10,000 cells per well and treated with different concentrations of vincristine as indicated (n = 3 except 100nm n = 2). (TIF) [file pone.0258140.s001.tif]

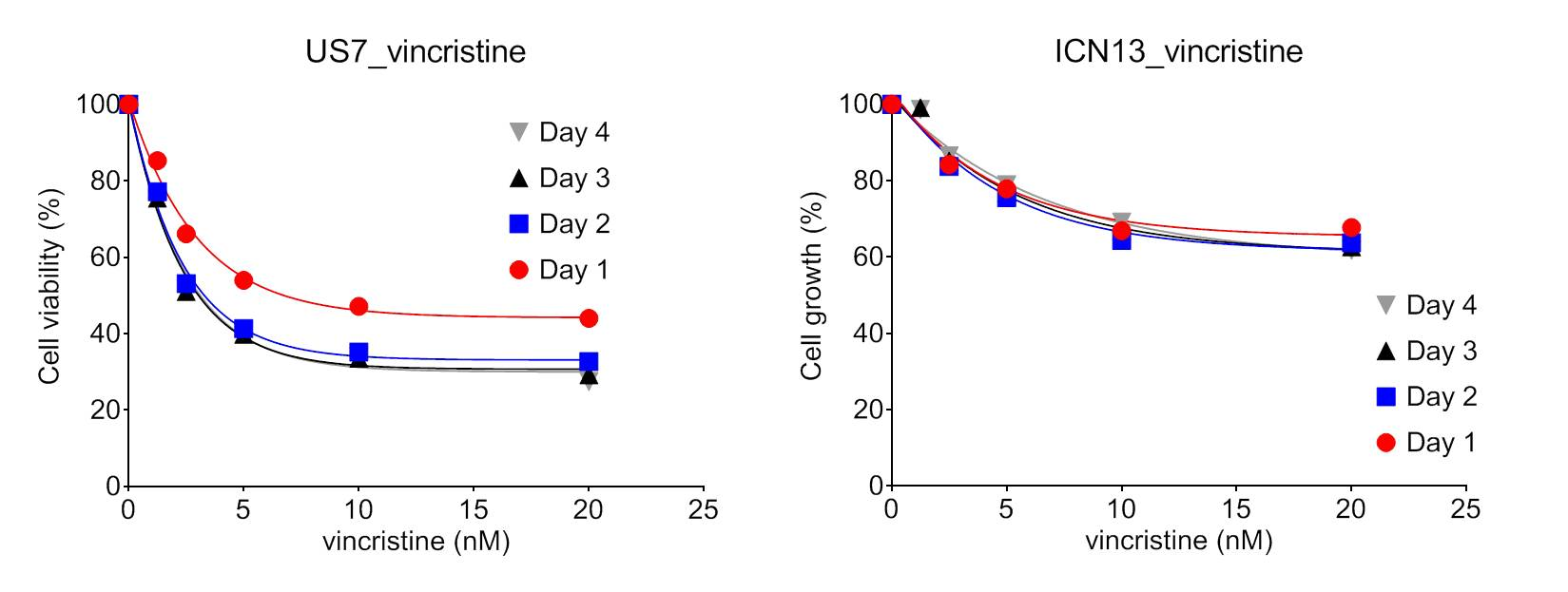

Supplement: S2 Fig — US7 and ICN13 BCP-ALL cells (5x10^4^) plated on irradiated OP9 stroma were treated with vincristine in the same experiment. Viability of cells was determined at 535–612 nm after a 5 hr incubation with 10% v/v Alamar blue. (TIF) [file pone.0258140.s002.tif]

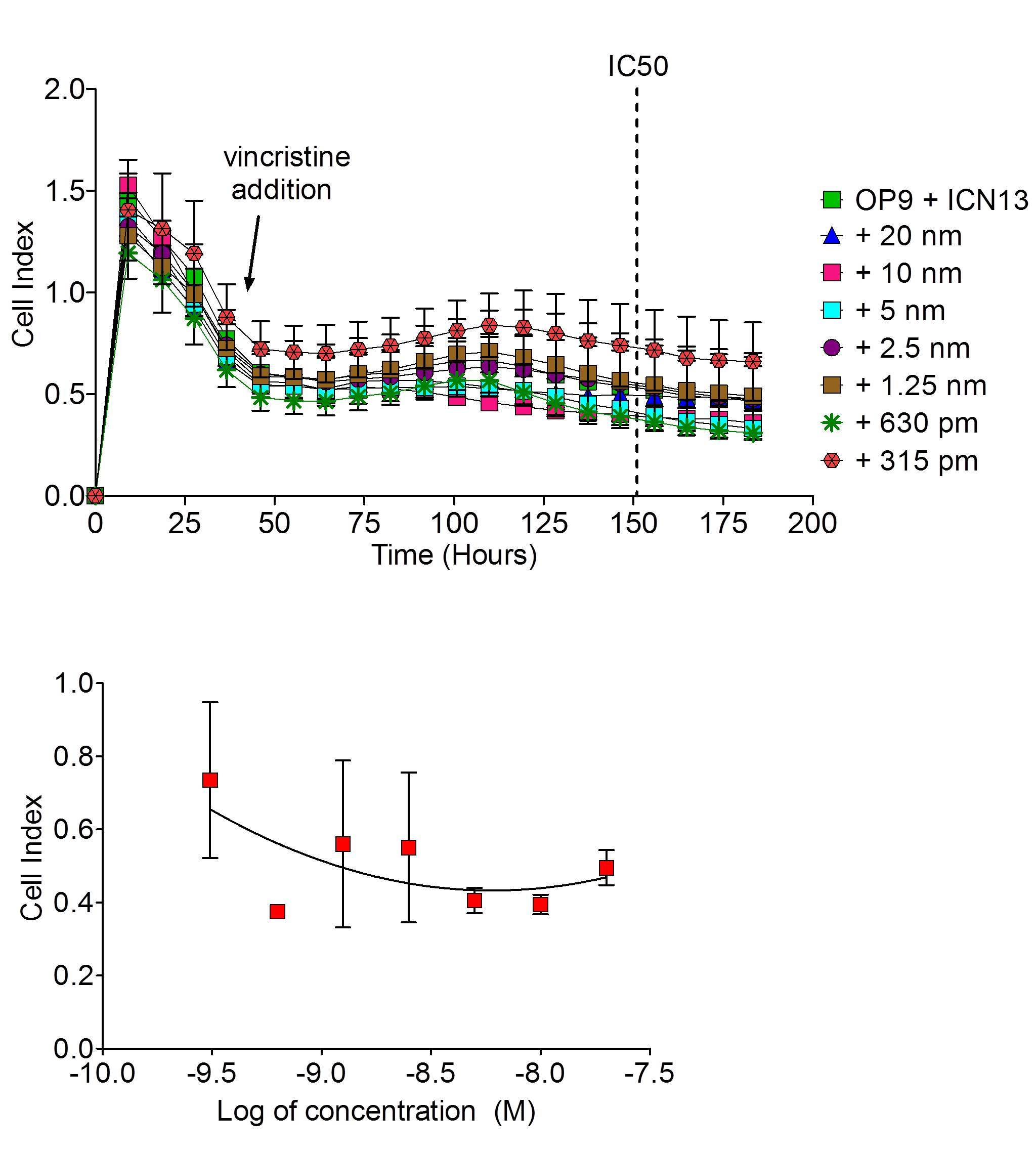

Supplement: S3 Fig — The dotted line indicates the time point used in the attempt to calculate IC50 (n = 3). (TIF) [file pone.0258140.s003.tif]

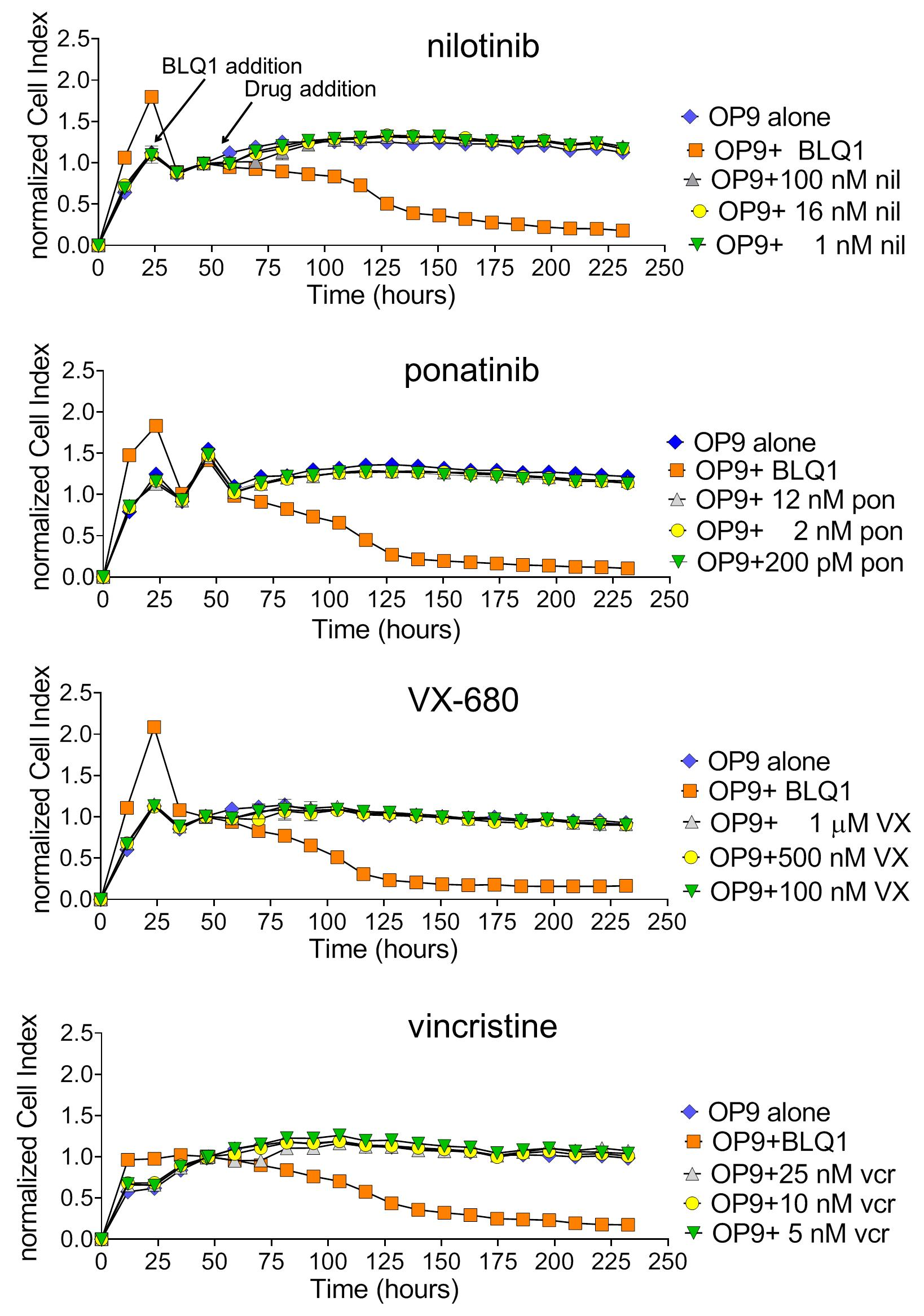

Supplement: S4 Fig — OP9 cells were mitotically inactivated, then plated at a concentration of 1x10^4^ cells and treated with the indicated concentrations of ponatinib, nilotinib, vincristine, and VX-680 as shown in the figure (n = 3). BLQ1 plated on OP9 cells is included as a reference plot. (TIF) [file pone.0258140.s004.tif]

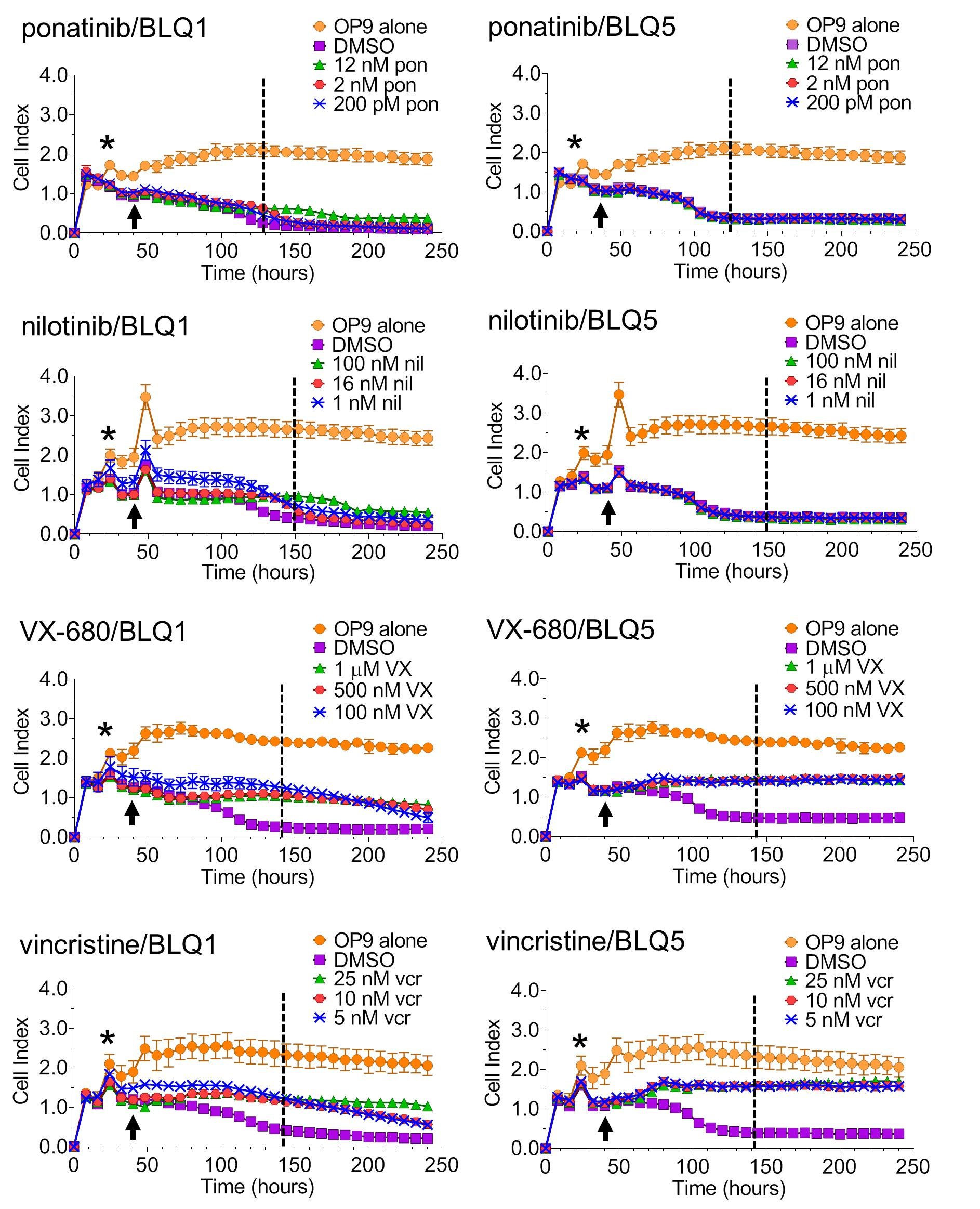

Supplement: S5 Fig — The percentage of displacement was determined approximately 125–150 hours after plating (n = 3). At this time point, the rate of change of the CI value was at its highest. Dotted lines show the time point used for measuring the % displacement in Fig 6A, 6B, bottom graphs. * identifies the time of BCP-ALL cell addition, whereas the arrowhead indicates the moment of addition of the drug. (TIF) [file pone.0258140.s005.tif]

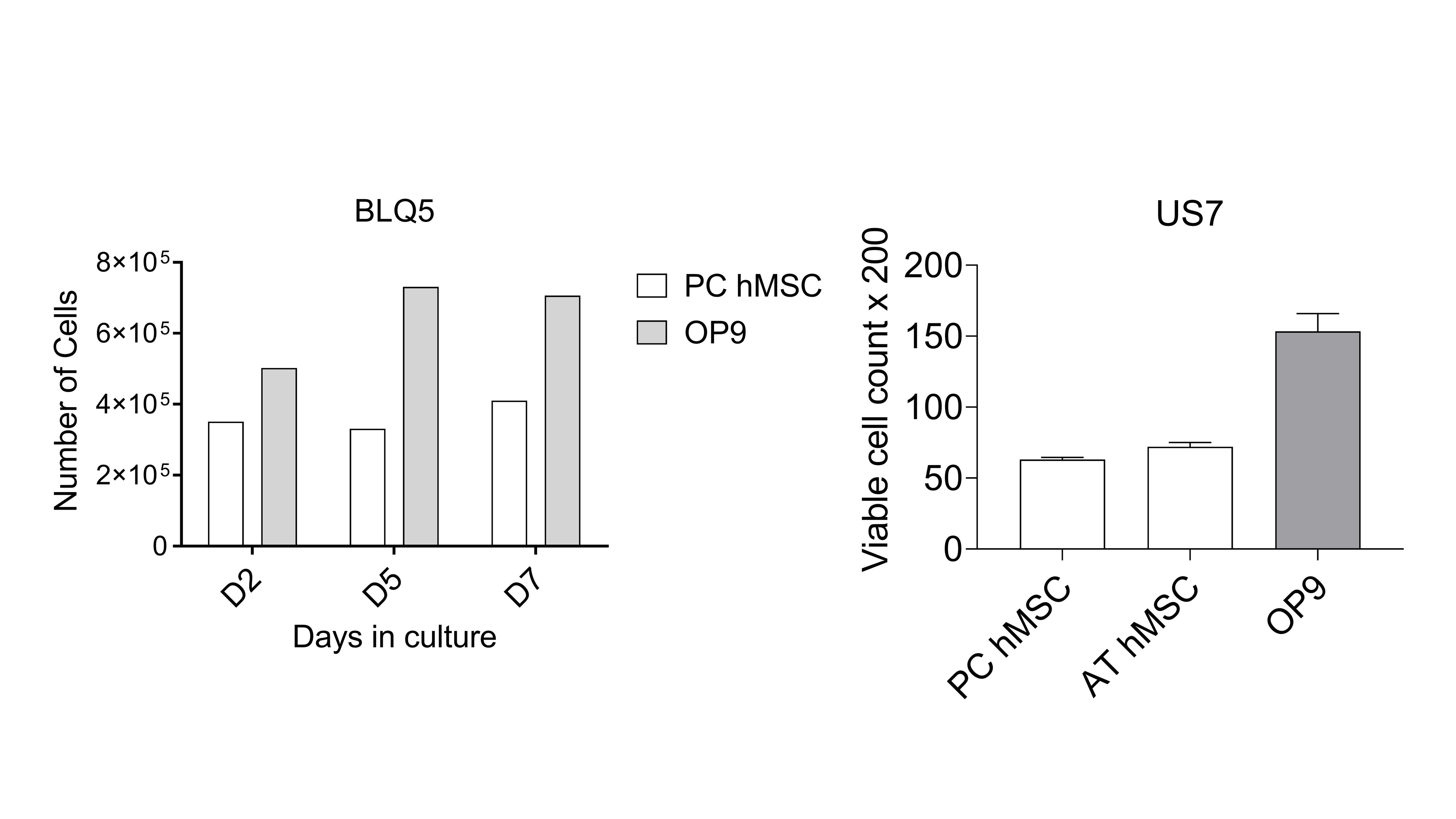

Supplement: S6 Fig — Human leukemia cells as indicated were grown on mitotically inactivated stromal cells and standard tissue culture plates as indicated (n = 2). Viable cell counts were determined by Trypan Blue exclusion. PC and AT, two different primary MSC sources. (TIF) [file pone.0258140.s006.tif]

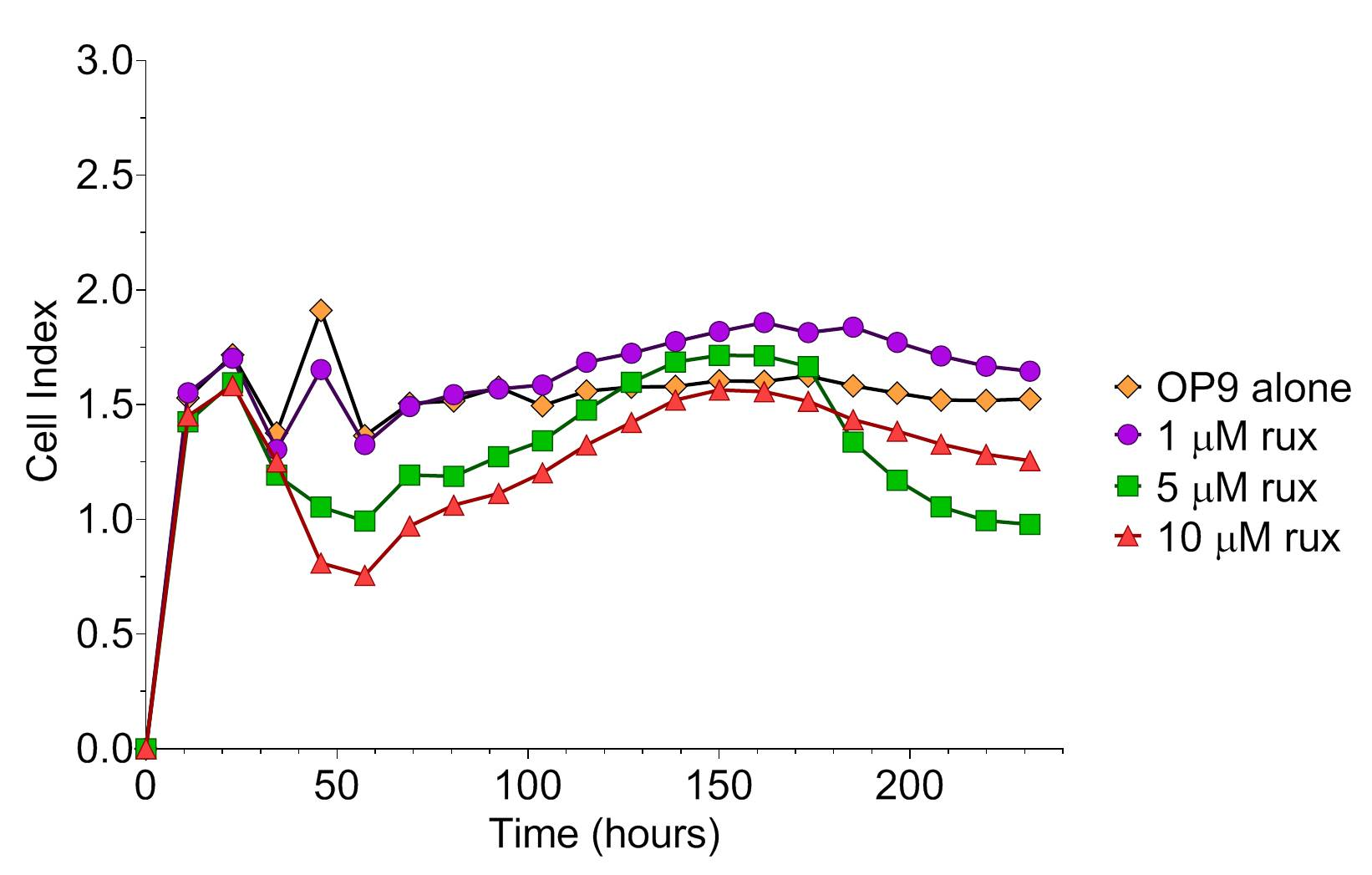

Supplement: S7 Fig — Mitotically inactivated OP9 cells were plated at a concentration of 10,000 cells per well and treated with different concentrations of ruxolitinib as indicated (n = 1). Note that the drug treatment only affected the CI/morphology of the cells up to 100 hours from the beginning of the experiment. (TIF) [file pone.0258140.s007.tif]
